# Stress-Induced Hyperglycaemia in Non-Diabetic Patients with Acute Coronary Syndrome: From Molecular Mechanisms to New Therapeutic Perspectives

**DOI:** 10.3390/ijms22020775

**Published:** 2021-01-14

**Authors:** Alessandro Bellis, Ciro Mauro, Emanuele Barbato, Antonio Ceriello, Antonio Cittadini, Carmine Morisco

**Affiliations:** 1 Unità Operativa Complessa Cardiologia con UTIC ed Emodinamica-Dipartimento Emergenza Accettazione, Azienda Ospedaliera “Antonio Cardarelli”, 80131 Napoli, Italy; abellis82@vodafone.it (A.B.); ciro.mauro1957@gmail.com (C.M.); 2 Dipartimento di Scienze Biomediche Avanzate, Università Federico II, 80131 Napoli, Italy; emanuele.barbato@unina.it; 3 Department of Cardiovascular and Metabolic Diseases, IRCCS Multimedica, Sesto San Giovanni, 20099 Milan, Italy; antonio.ceriello@hotmail.it; 4 Dipartimento di Scienze Mediche Traslazionali, Università Federico II, 80131 Napoli, Italy; antonio.cittadini@unina.it

**Keywords:** stress-induced hyperglycaemia, acute coronary syndrome, non-diabetic patient, insulin, GLP-1 receptor agonist, DPP-4 inhibitors, glifozins, multitargeted therapeutic strategy

## Abstract

Stress-induced hyperglycaemia (SIH) at hospital admission for acute coronary syndrome is associated with poor outcome, especially in patients without known diabetes. Nevertheless, insulin treatment in these subjects was not correlated with the reduction of mortality. This is likely due to the fact that SIH in the context of an acute coronary syndrome, compared to that in known diabetes, represents an epiphenomenon of other pathological conditions, such as adrenergic and renin-angiotensin system over-activity, hyperglucagonaemia, increase of circulating free fatty acids and pancreatic beta-cell dysfunction, which are not completely reversed by insulin therapy and so worsen the prognosis. Thus, SIH may be considered not only as a biomarker of organ damage, but also as an indicator of a more complex therapeutic strategy in these subjects. The aim of this review is to analyse the molecular mechanisms by which SIH may favour a worse prognosis in non-diabetic patients with acute coronary syndrome and identify new therapeutic strategies, in addition to insulin therapy, for a more appropriate treatment and improved outcomes.

## 1. Introduction

Stress-induced hyperglycaemia (SIH) is a severe and very common condition where blood glucose levels >140 mg/dL (7.78 mmol/L) occur during hospitalisation for critical diseases [1]. SIH usually resolves on recovery from the medical insult and before discharge from the hospital. SIH in the context of an acute coronary syndrome (ACS) is associated with a poor outcome in patients with or without known diabetes [2,3]. In particular, Capes et al. showed that the difference of intra-hospital mortality recorded between hyperglycaemic and normoglycaemic diabetic patients with ACS (1.7-fold) was lower than the difference of intra-hospital mortality between hyperglycaemic and normoglycaemic non-diabetic patients (3.9-fold) [4]. Consistently, Kosiborod et al. found a tight relation between high glycaemic levels at hospital admission and 30-day/1-year mortality in over 140,000 patients with ACS [5]. In this study, the authors found a stronger increase in mortality risk for patients without known diabetes versus the diabetic ones. Furthermore, Sinnaeve et al. could further establish that long-term glucometabolic dysregulation (fasting hyperglycaemia), in addition to SIH, increases the risk in ACS patients even in the absence of manifest diabetes [6]. Thus, SIH in ACS is an independent risk factor for cardiovascular (CV) mortality, especially in patients without known diabetes. Capes et al. suggested some explanations to justify the worse outcome of hyperglycaemic non-diabetic patients compared to hyperglycaemic diabetic patients [4]. These authors highlighted that it is difficult to precisely define SIH in diabetic subjects, because it is complex to establish their basal glycaemic values. Moreover, they underlined that diabetic patients may have more CV risk factors, and, for this reason, SIH and long-term glucometabolic dysregulation may have a minor impact. Finally, they remarked that a patient with known diabetes is more likely to receive insulin therapy, although almost two-thirds of the patients hospitalised for ACS with hyperglycaemia at admission showed impaired glucose tolerance (IGT) or a newly diagnosed diabetes at three months after discharge [7,8].

Another possible explanation of why hyperglycaemia is worsening the prognosis more in ACS people without diabetes than in those with diabetes is related to the evidence that acute hyperglycaemia promotes oxidative stress, which, in turn, can induce tissue injury [9,10] and may lead to bad CV outcomes [11,12]. Chronic hyperglycaemia induces antioxidant defences in cells and tissues [13,14]. Therefore, in presence of diabetes, there is a sort of “preconditioning phenomenon”, an increase of the antioxidant defences, which might better protect the tissues from the oxidative stress induced by acute hyperglycaemia, a condition that, of course, is not present in people without diabetes.

In last years, it has been questioned whether SIH really affects the outcome of ACS non-diabetic patients. In fact, most of data about SIH in ACS non-diabetic patients come from old clinical trials not including more recent anti-platelet drugs (prasugrel and ticagrelor) and modern drug-eluting stent (DES) platforms (at lower risk of restenosis compared to bare metal stents and first-generation DES). These findings may have determined an overestimation of SIH impact on the outcome of non-diabetic ACS patients. However, it has been recently reported that in ST-elevation myocardial infarction (STEMI) patients discharged alive, SIH is significantly associated with worse long-term prognosis in the non-diabetic population [15]. Thus, glucose normalization during ACS seems to be always beneficial.

The aim of this review is to analyse the molecular mechanisms by which SIH may favour a worse prognosis in non-diabetic patients with ACS and try to identify the rationale of new therapeutic strategies, in addition to insulin therapy, in order to improve the outcomes in this setting.

## 2. SIH in the Context of Acute Coronary Syndromes

SIH in ACS derives from combination of impaired insulin secretion to overcome the hyperglycaemic effects of counter-regulatory hormones and tissue insulin resistance (IR). In particular, it is well known that pancreatic beta-cell dysfunction affects insulin release [7,16], thereby increasing glucagon and glucose levels in acute ill conditions, such as ACS [17,18]. Consistently, the sympathetic nervous system activation induced by ACS further stimulates glucagon release [19,20]. In addition, dysregulation of sympathetic nervous and renin–angiotensin–aldosterone systems (RAAS) [21], occurring during ACS and resulting in increased stimulation of both adrenergic receptors (AR) and angiotensin II (AT-II) receptors, seems to be the principal mechanism accounting for acute IR.

### 2.1. Pancreatic Beta-Cell Dysfunction and Increased Glucagon Levels

SIH during ACS may be due to pancreatic beta-cell dysfunction. In the Glucose Tolerance in Patients with Acute Myocardial Infarction (GAMI) study, IR did not differ between patients with ACS plus newly discovered glucose disturbances and matched controls. Conversely, the pro-insulin levels were significantly increased in patients compared to control subjects indicating impaired beta-cell function among these subjects [7,16].

In ACS, glucagon levels may grow up to five-fold the upper normal value. Under physiological conditions, glucagon secretion from pancreatic alpha-cells is regulated by fluctuations in plasma glucose through the autonomic nervous system, circulating hormones, glucagon-like peptide-1 (GLP-1), and insulin [22]. Because of beta-cells dysfunction, insulin may be absent or low in ACS when a patient is often not eating normal amounts of food and when stress levels of catecholamines, which suppress insulin secretion, are very high [23,24]. There is evidence that most of the inhibitory effect of insulin on glucagon secretion is mediated by paracrine effects within the pancreatic islets [25]. However, it has been postulated that administration of supraphysiological insulin levels to suppress glucagon secretion will probably not be sufficient to reach normal glycaemic values in hyperglucagonaemic patients with SIH [26].

### 2.2. Sympathetic Nervous System

Sympathetic nervous system plays a double role in SIH. On the one side, epinephrine has been shown to directly stimulate glucagon secretion [27,28,29]. Under extreme stress conditions, such as after acute myocardial infarction (AMI) and cardiac arrest, epinephrine levels can markedly increase and may have a greater effect on glucagon secretion in the critically ill patient [30,31].

On the other side, it has been demonstrated that stimulation of β-ARs can evoke IR, through the stimulation of several serine/threonine kinases that interfere with the insulin signalling by phosphorylating insulin receptor substrate-1 (IRS-1) through different mechanisms and kinetics, depending on the receptor subtype expressed. For instance, β3-AR stimulation in adipocytes impairs insulin signalling within few minutes [32], whereas, in cardiac myocytes, stimulation of β1-AR has a biphasic effect on insulin-stimulated glucose uptake, with an initial additive action, followed by an inhibitory one [33], due to an impairment of MAPK phosphatase-1 expression [34].

Moreover, the initial pain-related burst of catecholamine release: (1) mobilizes free fatty acids (FFAs) from adipose tissue; (2) acutely inhibits the release of insulin from the pancreas; and (3) causes hyperglycaemia [35]. The elevated plasma FFAs are preferentially oxidized by skeletal and cardiac muscle, hence impairing the uptake and oxidation of glucose [36] and directly contributing to IR [37,38]. Thus, hyperglycaemia is the consequence of both the high circulating FFAs and catecholamine discharge. This latter additionally promotes hepatic glycogenolysis. At a mitochondrial level, FFAs excess induces low ratios of ATP production/oxygen consumption and excess production of electrons, which are transferred to molecular oxygen without ATP production [38]. The following increased production of free radicals can inactivate the IRS-1 by phosphorylating serine residues, thereby directly promoting IR [38].

### 2.3. Renin–Angiotensin System (RAAS)

In vivo and in vitro studies have shown that the stimulation of AT-II also induces IR. Interventional studies have documented that angiotensin converting enzyme (ACE)-inhibitors [39] and angiotensin receptor blockers (ARBs) [40] reduce the incidence of type 2 diabetes mellitus (T2DM). Therefore, overactivity of RAAS observed in CV diseases is likely to impair insulin signalling and contributes to IR. Actually, AT-II acting through the AT-I receptor inhibits the actions of insulin in vascular tissue, in part by interfering with insulin signalling through phosphatidyl-inositol-3 kinase (PI3K) and downstream Akt signalling pathways via generation of reactive oxygen species (ROS) [41]. ROS are important intracellular second messengers that regulate several downstream signalling molecules, such as phosphotyrosine phosphatases (PTPases) and protein tyrosine kinases. PTPases are key regulators of tyrosine phosphorylation-dependent signalling, and tyrosine dephosphorylation by PTPases may have a pivotal role in AT-II-induced IR. Several PTPase have been implicated in the AT-II-induced dephosphorylation of insulin receptor. In particular, PTPase-1B seems to play a critical role in insulin action [42]. Other studies have shown that ROS generation is involved in the AT-II-evoked IR. In this regard, it has been documented that, in vascular smooth muscle cells isolated from rat thoracic aorta, AT-II profoundly decreases IRS-1 protein levels via ROS-mediated phosphorylation of IRS-1 on Ser307 and subsequent proteasome-dependent degradation [43,44]. The key role of ROS in the pathogenesis of AT-II-induced IR has also been confirmed by in vivo studies. In particular, in rats, chronic infusion of AT-II reduced insulin-induced glucose uptake during hyperinsulineamic–euglycaemic clamp and increased plasma cholesteryl ester hydroperoxide levels, indicating an enhanced oxidative stress. Treatment with tempol, a superoxide dismutase mimetic, normalized plasma cholesteryl ester hydroperoxide levels. In addition, tempol restored insulin sensitivity in AT-II-infused rats, as well as enhanced insulin-induced PI3K activation, suggesting that the development of AT-II-induced IR can be antagonized by removing the oxidative stress [45]. On the other hand, in endothelial cells, AT-II induces IR through the phosphorylation of IRS-1 at Ser312 and Ser616 via JNK- and ERK 1/2-dependent pathways, respectively. This event impairs the interaction of IRS-1 with the p85 regulatory subunit of PI3K and compromises the insulin-mediated vasodilatory effect [46].

## 3. Detrimental Effects of SIH in Acute Coronary Syndrome

Multiple pathophysiological studies have demonstrated that SIH may have a direct detrimental effect on ischemic myocardium through multiple mechanisms. In particular, Kersten et al. showed an impairment of collateral circulation associated with increased infarct size in the setting of severe hyperglycaemia [47,48]. Moreover, hyperglycaemic patients with STEMI have lower rates of spontaneous reperfusion [49]. Coronary microvascular dysfunction was further demonstrated in hyperglycaemic patients with STEMI undergoing to reperfusion. Specifically, Iwakura et al. showed a higher incidence of the no-reflow phenomenon in patients with elevated glucose levels after successful reperfusion [50]. Consistently, thrombus aspiration during primary percutaneous intervention (pPCI) for STEMI reduced clinical outcomes in hyperglycaemic patients, whereas it did not in normoglycaemic ones [51]. Human studies have also linked elevated glucose levels with endothelial dysfunction, as measured by endothelium-mediated brachial artery vasodilation, in which the level of endothelial dysfunction was correlated with the level of hyperglycaemia [52].

Furthermore, several studies have demonstrated that hyperglycaemia is associated with a prothrombotic state. Acutely, hyperglycaemia in rats evokes the decrease of tissue plasminogen activator activity and increases plasminogen activator inhibitor levels [53]. SIH induces a shortening of the half-life of fibrinogen and platelet aggregation and results in increased levels of fibrinopeptide A, prothrombin fragments and factor VII. All these phenomena suggest increased activation of prothrombotic factors [54,55,56]. In addition, it is also reasonable to hypothesize that the detrimental effects of SIH in ACS can be amplified by the genetic variants of the coagulation factors resulting in a vicious circle that amplify the platelet aggregation and the prothrombotic state [57]. Higher glucose levels have been shown to be associated with increased markers of vascular inflammation. Both in vitro and in vivo studies have linked hyperglycaemia with elevated levels of C-reactive protein (CRP), interleukin-6 (IL-6), and TNF-α [58,59]. TNF-α has been shown to extend infarct size in experimental settings and to induce cardiac myocyte apoptosis [60,61]. In vitro and in vivo studies also demonstrated induction of the proinflammatory transcription factor NF-κB in a setting of elevated glucose [62,63]. This inflammatory response evokes a detrimental effect on injured myocardium [64]. Moreover, glucose ingestion in healthy human volunteers is associated with increased production of other proinflammatory factors, such as activator protein 1 and early growth response 1, and increased expression of the genes regulated by them, including the genes for matrix metalloproteinases-2 and -9 and tissue factor (TF) [65].

Interestingly, data from human studies suggest that acute fluctuations in glucose levels may have an even more powerful impact on oxidative stress than chronic and sustained hyperglycaemia [66]. Further results suggest that hypoglycaemia may play an important role in the vascular damage [67]. During hypoglycaemia, oxidative stress is produced at the mitochondrial level [68], similarly to in hyperglycaemia [69]. In particular, there is experimental evidence that free radical production does not only rise during hypoglycaemia, but especially does during glucose reperfusion after hypoglycaemia [70]. The adverse effects of hyperglycaemia following hypoglycaemia were analyzed in a human study, comparing normal control individuals and people with type 1 diabetes mellitus (T1DM) [71]. Recovery with normoglycaemia is accompanied by a significant improvement in endothelial dysfunction, oxidative stress and inflammation, which are affected by hypoglycaemia. However, a period of hyperglycaemia after hypoglycaemia worsens all of these parameters, an effect that persists even after the additional 6 h of normoglycaemia. This effect is partially counterbalanced when hyperglycaemia after hypoglycaemia is accompanied by the simultaneous infusion of vitamin C, thereby suggesting that, when hyperglycaemia follows hypoglycaemia, an ischemia–reperfusion-like effect is produced.

Finally, higher glucose levels exert a detrimental effect during ACS because they are also associated with higher FFAs concentrations leading to a direct myocardial injury [72,73]. When circulating FFAs are high enough to exceed the tight binding sites on the albumin, they determine an increased enzyme release in the ischemic myocardium [74]. Such FFA levels are reached in the early hours of ACS [75,76]. The leading mechanisms to explain such effects are the membrane-damaging detergent properties of FFAs [77] and an increased oxygen demand due to mitochondrial uncoupling, which underlies the FFAs-induced increase in myocardial oxygen consumption and local heat production [78]. Excess circulating FFAs may also increase myocardial diastolic dysfunction [79].

## 4. Results of Insulin Therapy for SIH in Acute Coronary Syndrome

The analysis of mechanisms by which hyperglycaemia induces detrimental effects on ischemic myocardium suggests an early start of insulin treatment in non-diabetic patients with ACS and SIH at admission. In fact, insulin exerts a lot of potential benefits on ischemic myocardium: (a) anti-inflammatory action, through the inhibition of the activity of nuclear factor-kB (NF-kB) and reduction of major chemotactic protein (ICAM-1) and CRP; (b) anti-thrombotic action, mediated by the inhibition of TF and plasminogen activator inhibitor; (c) vasodilator effects, through platelet inhibition and increase in nitric oxide (NO) and cyclic AMP (cAMP) release; (d) glucose lowering action; (e) anti-apoptotic effects; and (f) anti-oxidant effects, mediated by inhibition of nitric oxide synthase (NOS) activity and consequent reduction of NO^-^ free radicals production [80]. Nevertheless, interestingly, insulin therapy did not reduce mortality in dedicated randomized clinical trials (RCTs).

RCTs evaluating the effects of SIH pharmacological treatment in ACS patient can be divided into two groups: (1) studies comparing a tighter glycaemic control versus standard therapy (Table 1); and (2) studies testing the effects on outcome of the glucose-insulin-potassium (GIK) versus standard therapy, independently on obtained glycaemic values (Table 2).

### 4.1. Tighter Glycaemic Control Versus Standard Therapy

In the first group, the DIGAMI 1 trial studied the effects of intensive in-hospital insulin treatment (insulin-glucose infusion for at least 24 h followed by subcutaneous multiple dose insulin regimen) versus the conventional treatment in 620 patients with ACS and established diabetes and/or admission glucose of >200 mg/dL (11 mmol/L) [81]. Better glucose control was achieved in the intensive insulin therapy arm. A significant mortality benefit was seen in the intervention arm at both the 1- and 3.4-year follow-up points [82]. In addition, a 20-year follow-up of DIGAMI 1 trial showed that median survival time was 7.0 years in the intensified glycaemic control group and 4.7 in those in the standard group. Thus, the effect of intensified glycaemic control was apparent during eight years after randomisation, increasing survival by 2.3 years [83]. The DIGAMI 1 study was the only RCT of glucose control in ACS which has achieved a significantly lower glucose level in the intervention arm compared to the control arm. It is also the only RCT which has demonstrated a survival benefit associated with better glucose control. On the contrary, DIGAMI 2 trial attempted to study three alternative treatment regimens: acute insulin-glucose infusion followed by insulin-based long-term glucose control; insulin-glucose infusion followed by standard glucose control on discharge; and routine metabolic management in both inpatient and outpatient settings [84]. There were no differences in outcomes among the 1253 randomized ACS patients. This may be attributable to the similar short-term glucose control and identical longer-term glucose control obtained among the three groups. Most importantly, the longer-term fasting glucose target of 90–126 mg/dL (5–7 mmol/L) was never achieved in the intensive-treatment group. Thus, despite its intent, DIGAMI 2 ended up comparing different insulin-treatment strategies, not different intensities of glucose control. Furthermore, similar to the DIGAMI 1 trial, DIGAMI 2 did not include hyperglycaemic patients without previously known diabetes, which is the group at highest risk of glucose-associated death. 

Successively to DIGAMI studies, to avoid recruitment bias, randomized clinical studies were designed to evaluate the effect of intensive insulin therapy in a population comprising a large percentage of non-diabetic patients with SIH at admission for ACS. The HI-5 study was the first RCT of intensive insulin infusion that included hyperglycaemic ACS patients without previously established diabetes [85]. Patients assigned to the intensive insulin-infusion arm received standard insulin and dextrose infusion that was then adjusted to maintain glucose levels between 72 and 180 mg/dL (4 and 10 mmol/L). Patients in the conventional arm received their baseline diabetes medications (including subcutaneous insulin); additional short-acting subcutaneous insulin was permitted for those with a glucose level >288 mg/dL (16 mmol/L). There were only 244 patients randomized in the study. There was no difference in mortality rates among the groups during hospitalization or at three or six months. There were, however, statistically and clinically significant reductions in post-myocardial infarction heart failure (HF) during hospitalization (10% absolute risk reduction) and in reinfarction at three months (3.7% absolute risk reduction). These data allow to hypothesize that the tight control of glycaemia prevents the post-infarction left ventricular remodelling (LVR), which still affects the 30% of ACS patients [86].

In addition, smaller clinical studies gave controversial results. In particular, some of these demonstrated a positive effect of tight glycaemic control, associated to the achievement of glucose target, compared to standard therapy on myocardial injury in hyperglycaemic patients addressed to coronary artery bypass graft (CABG) [87,88] or pPCI [89,90]. Conversely, others did not evidence a significant statistical difference in achieved target glycaemic values, as well as in 90-day mortality or extension of myocardial injury between two therapeutic strategies [91,92].

Thus, the results of these RCTs demonstrate that a tighter glycaemic control leads to a better prognosis, but this aim is very difficult to achieve with insulin alone. 

### 4.2. GIK Versus Standard Therapy

About RCTs included in the second group, the aim of investigators was to demonstrate the superiority of GIK versus standard therapy, independently on achievement of a specific glycaemic target. In ECLA-GIK trial, were enrolled 407 patients with suspected AMI, admitted within 24 h after the onset of symptoms [93]. In a ratio of 2:1, 268 patients were allocated to receive GIK and 139 to receive control. A trend toward a non-significant reduction in major and minor in-hospital events was observed in patients allocated to GIK.

The GIPS-I study showed a clinical benefit of GIK therapy in STEMI patients without signs of HF [94]. On the contrary, the GIPS-II trial, designed to confirm the positive results of GIPS-I in STEMI patients receiving reperfusion therapy, did not show any trend toward a therapeutic benefit [95]. Similarly, the CREATE-ECLA [96] and the OASIS-6 GIK [97] trials have convincingly shown that GIK infusion has no effect on mortality, cardiac arrest or cardiogenic shock in ACS.

Finally, the IMMEDIATE study showed that, among patients with suspected ACS, out-of-hospital administration of intravenous GIK, compared with glucose placebo, did not reduce progression to myocardial infarction [98]. Compared to placebo, GIK administration was not associated with an improvement in 30-day survival but was associated with lower rates of the composite outcome of cardiac arrest or in-hospital mortality.

Thus, these RCTs failed to determine mortality rate reduction in patients with SIH at admission for ACS. This finding could be dependent by high potassium concentrations in GIK solutions. In fact, a combined evaluation of all studies showed that such therapy might even be harmful in the early phase of ACS, due to hyperkalaemia or the fluid challenge [99].

### 4.3. Clinical Remarks of RCTs with Insulin

The real common denominator between these two study groups was that insulin therapy did not significantly reduce the glycaemic values. It is important to remark that glucose normalization during ACS is beneficial, irrespective whether the patients receive insulin or not [100]. In fact, Svensson et al. showed that patients whose lowest blood glucose readings during hospitalization for ACS were >120 mg/dL (6.67 mmol/L) had a 46% increase in relative risk of 30-day mortality compared with patients whose lowest glucose values were between 56 and 119 mg/dL (3.11 and 6.61 mmol/L). This relationship was present regardless of admission glucose values [101]. Furthermore, Goyal et al. evaluated the effect of the change between 24 h and admission glucose levels and death [102]. They found that an increase in glucose values during the first 24 h of hospitalization was associated with higher 30- and 180-day mortality rates, whereas a fall in the glucose level was associated with improved survival: this relationship was present in patients without diabetes but not in those with diabetes. Importantly, this study was not able to differentiate between spontaneous and insulin-mediated decreases in glucose values.

In general, the major limitation of these studies is that they tested the effects of insulin treatments (intensive versus conventional) in a population with IR state: this fact explains the modest results achieved in this clinical context. Such limitation, however, highlights the need to evaluate therapies capable to contrast the development of IR, rather than different insulin treatments. 

## 5. Therapeutic Strategies for Improving Prognosis

**Figure 1:**
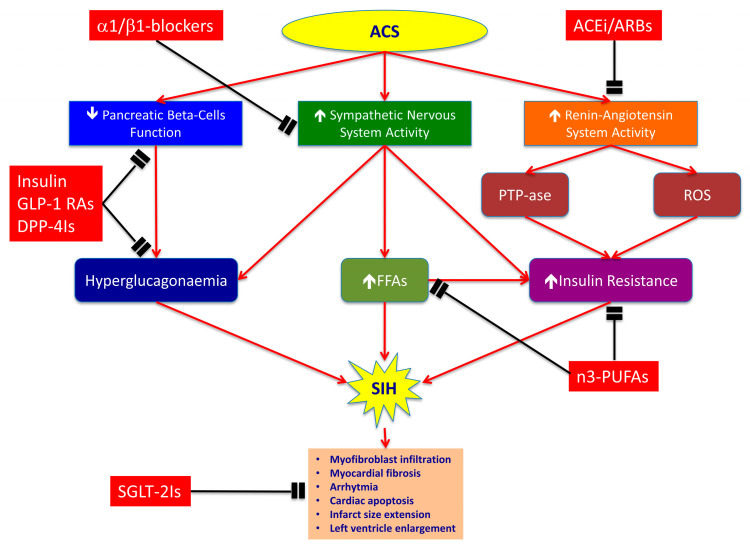
Mechanisms of stress-induced hyperglycaemia (SIH) during acute coronary syndrome (ACS) and complementary effects of each pharmacological class. ACS determines SIH by decreasing insulin release through pancreatic beta-cells dysfunction, and by inducing insulin resistance (IR) through overactivation of sympathetic nervous and renin-angiotensin systems. The insulin decrease and adrenergic overdrive provoke hyperglucagonaemia and increase of free fatty acids (FFAs) plasmatic levels, thereby leading to hyperglycaemia. Contemporarily, renin–angiotensin–aldosterone system hyper-stimulation induces IR through the activation of phosphotyrosine phosphatases (PTPases) and reactive oxygen species (ROS) formation. Selective α1/β1-blockers, such as nebivolol, antagonize adrenergic overdrive and have vasodilatory properties that are mediated by stimulation of nitric oxide (NO) release from endothelial cells. ACE inhibitors (ACEi) and angiotensin receptor blockers (ARBs) inhibit PTPases activity and ROS formation, thereby reducing IR. The n3-polyunsaturated fatty acids (n3-PUFAs) improve cholesterol levels and insulin sensitivity compared to diets high in saturated fats. Dipeptidyl peptidase-4 inhibitors (DPP-4Is) and glucagon-like peptide-1 receptor agonists (GLP-1 RAs) improve pancreatic beta-cells function and reduce glucagon release by alfa-cells, thereby lowering HbA1c levels. Finally, sodium-glucose co-transporters 2 inhibitors (SGLT-2Is) have cardiovascular benefits mostly unrelated to the extent of glucose lowering. They could involve effects on haemodynamic parameters, such as reduced plasma volume, and direct effects on cardiac metabolism and function, such as myocardial fibrosis, cardiac apoptosis and infarct size enlargement. Red lines indicate activation pathways; black lines indicate inhibition pathways; ↑indicates an increase; ↓indicates a decrease.

Actually, it clearly appears that treating SIH in non-diabetic patients with ACS requires more than insulin therapy. In particular, we need drugs counteracting the adrenergic system overdrive, the RAAS overactivity, the FFAs concentration increase and the impairment of beta-cell function, all contributing to IR and to extension of myocardial injury. In this section, we discuss the utility of β -blockers, ACE-inhibitors, ARBs, polyunsaturated fatty acids (PUFAs) and new hypoglycaemic agents (GLP-1 receptor agonists, inhibitors of dipeptidyl peptidase-4 and sodium-glucose co-transporters 2 inhibitors) to improve the prognosis in non-diabetic patients with SIH at admission for ACS (Figure 1).

### 5.1. β-Adrenergic Blockers

To tackle the β -adrenergic stimulation that causes both FFAs elevation and IR, it would logically require early β-blockade, a procedure known to reduce plasma FFAs uptake by the failing myocardium [103] and to lessen FFAs accumulation in the ischemic-reperfused heart [104]. However, routine early intravenous β-blockade may have adverse hemodynamic consequences including cardiogenic shock and hypotension [105]. On the other hand, giving a β-blocker later, once hemodynamic stability has been attained, it would be missed the crucial first 3 h when reduction of infarct size is most likely to be achieved [106].

Furthermore, abnormalities of glucose, insulin, and lipid and carbohydrate metabolism have been reported frequently during treatment with conventional β-blockers [107]. In a meta-analysis of 22 clinical trials mostly in patients with hypertension, Elliott and Meyer documented the odds ratio of new-onset T2DM compared with placebo to be highest with diuretics and β-blockers (included studies using predominantly atenolol or metoprolol) and lowest with ARBs and ACE-inhibitors [108]. 

Conversely, clinical trial data suggest that vasodilating β-blockers, such as carvedilol, a β1/β2-antagonist with α1-blocking activity, and nebivolol, a highly β1-selective agent with NO-mediated vasodilatory effects, have neutral or even beneficial metabolic effects, and thus can be potentially useful in patients with ACS [109,110]. The GEMINI trial compared carvedilol with the non-vasodilating agent metoprolol in 1235 patients with hypertension and T2DM [111]. All patients received background therapy with a RAAS blocker. The results show that the addition of carvedilol, compared to metoprolol, had a favourable effect on glycaemic control, IR, microalbuminuria and body weight. The differences observed between metoprolol and carvedilol in the presence of an ACE-inhibitors or an ARB are important and suggest that the metabolic effects of β-blockers remain clinically significant even in the context of RAAS blockade [112]. Similarly, the highly β1-selective agent nebivolol has vasodilatory properties that are mediated by its stimulation of NO release from endothelial cells [113,114]. Studies comparing nebivolol with atenolol [115] and with metoprolol [116] showed neutral effects of nebivolol on insulin sensitivity, whereas both β-blockers increased IR.

Thus, combination therapy of β1-blockers and RAAS agents has the potential to cause further favourable metabolic responses.

### 5.2. ACE Inhibitors and ARBs

It has been reported that AT-II increases hepatic glucose production and decreases insulin sensitivity, whereas ACE-inhibitors and ARBs increase insulin sensitivity [117,118]. Although the beneficial impacts of RAAS inhibitors on the prevention of new-onset T2DM have been reported in several clinical trials involving patients with hypertension, chronic HF or stable coronary artery disease [119,120,121], data are limited in the secondary prevention for ACS.

In a study that analyzed the relationship between baseline glucose and left ventricular enlargement (LVE), it was demonstrated that LVE correlated significantly with baseline hyperglycaemia, failed reperfusion by ECG criteria and no use of ACE-inhibitors or ARBs and aspirin [122]. On the contrary, a history of previous diabetes did not correlate significantly with LVE at six months. 

A further study revealed that SIH after the onset of ACS may be an independent predictor of de novo-T2DM development in non-diabetic ACS patients and also suggested that inhibition of the RAAS may attenuate the risk of de novo-T2DM in the secondary prevention setting after ACS with SIH [123]. This observation is consistent with our recent observations. In particular, we found, in a different clinical context, that the severity of coronary atherosclerosis is predictive of new onset of T2DM [124], and that there is a strong association between IR and severity of coronary artery disease [125].

Thus, the administration of drugs acting on RAAS seems to be useful in reducing LVR and the onset of de novo-T2DM in patients with SIH at hospital admission for ACS, but further investigations are warranted to examine the beneficial impacts and mechanisms of RAAS inhibitors in order to obtain this result.

### 5.3. Mono- and Poly-Unsaturated Fatty Acids

To directly reduce circulating FFAs, nicotinic acid or its derivatives and insulin may be used. In this regard, there are several pioneering studies, showing that, in the intact dog heart, FFAs-induced “oxygen-wastage” was reduced by about 40% either by nicotinic acid or by intravenous glucose [126]. Antilipolytic therapy given to patients with early-phase ACS by a nicotinic acid analogue brought down FFAs levels and lessened electrocardiographic signs of ischemia, but was not subjectively acceptable because of the high incidence of flushing and hypotension [127]. GIK infusions given to patients substantially decreased FFAs levels [128], with less dramatic FFAs-lowering effects when low-dose GIK was used [76]. A similar result was obtained with intravenous glucose [75]. Ideally, circulating FFAs would be reduced to the estimated threshold for myocardial FFAs uptake (about 200 µmol/L) [128]. However, in a perfused rat heart with coronary ligation, decreasing the perfusate FFAs from 1.5 to 0.5 mmol/L, without any added glucose or insulin, reduced enzyme release by about half [74], thereby demonstrating that it is not essential to reduce FFAs levels down to the hypothetical threshold for FFAs uptake by the myocardium, and that reduction of FFAs by itself lessens cellular ischemic damage.

As neither nicotinic acid analogues nor GIK infusions are ideal strategies to reduce plasma FFAs in patients, because of their adverse effects (flushing and hyperglycaemia), administration of mono- and poly-unsaturated fatty acids (MUFAs and PUFAs) should be considered. Although MUFAs and/or PUFAs improve cholesterol levels and insulin sensitivity compared to diets high in saturated fats, RCTs have not consistently demonstrated benefits of PUFAs and MUFAs in prevention or reversal of CV disease. Moreover, intake of n-6 PUFAs alone without a concomitant increase in n-3 PUFAs not only fails to reduce CV risk, but also may increase it [129,130]. Both n-3 and n-6 PUFAs are precursors of eicosanoids that serve as signalling molecules, but the major eicosanoid metabolites and their functional endpoints differ. Eicosanoids derived from n-6 PUFAs promote inflammation and development of atherosclerosis, whereas those from n-3 PUFAs are anti-inflammatory and protect against atherosclerosis [131,132]. Furthermore, n-3 PUFAs have been observed to increase the capacity for skeletal muscle fatty acid oxidation and improve insulin sensitivity.

To date, few studies have addressed the ability of dietary fatty acids to reverse established glucose intolerance and vascular dysfunction. The majority of studies have assessed dietary fatty acids in a preventative role, but the ability to reverse existing metabolic dysfunction may be more clinically relevant. A study was conducted in mice fed with a high saturated fat diet (60% kcal from lard) for 12 weeks before substituting half the lard with n-3 PUFA-enriched menhaden oil (MO), n-6 enriched safflower oil (SO) or MUFA-enriched olive oil (OO) for an additional four weeks [133]. The authors noted that, after 12 weeks of saturated fat diet, body weights were elevated and glucose tolerance was abnormal compared to mice on a controlled diet (13% kcal lard). Nevertheless, diet substituted with MO restored basal glucose levels, glucose tolerance and indices of insulin signalling (phosphorylated Akt) at normal levels, whereas restoration was limited for SO and OO substitutions. Although dilation to acetylcholine was reduced in arteries from mice on high saturated fat diet with OO and SO diets compared to normal diet, dilation to acetylcholine was fully restored and constriction to phenylephrine was reduced in MO-fed mice compared to normal. They concluded that short-term enrichment of an ongoing high fat diet with n-3 PUFA rich MO, but not MUFA rich OO or n-6 PUFA rich SO, reverses glucose tolerance, insulin signalling and vascular dysfunction.

Thus, it could be interesting to test introduction of n-3 PUFA-enriched MO in patients with ACS and hyperglycaemia at admission to evaluate its role in improving prognosis.

### 5.4. Glucagon-Like Peptide 1 Receptor Agonists and Dipeptidyl Peptidase-4 Inhibitors

The GLP-1, which is secreted in the gut in response to food ingestion, exerts a glucose-regulatory action via stimulation of insulin secretion (beta-cells) and suppression of glucagon (alfa-cells) in a glucose-dependent manner [134]. Moreover, GLP-1 decelerates gastric emptying, which was found to be associated with marked effects on post-meal glycaemic excursions [135]. GLP-1 is rapidly degraded by the enzyme dipeptidyl peptidase-4 (DPP-4) [134]. 

GLP-1 has beneficial effects by improving glucose and lipid metabolism, weight loss and endothelial function [136]. In addition to their glucose-lowering effect, the GLP-1 seems to protect pancreatic beta-cells by enhancing cell proliferation and differentiation, inhibiting apoptosis and stimulating insulin biosynthesis/secretion [137,138,139,140].

It has been recently found that endogenous circulating GLP-1 concentrations are elevated in patients with ACS and that increased GLP-1 levels are associated with CV events [141]. However, it appears unlikely that higher GLP-1 levels after ACS are detrimental and responsible for adverse outcomes and early death. In fact, a previous experimental work showed that the increased GLP-1 secretion in response to myocardial infarction was cardioprotective in mice by augmenting left ventricular contractility [142]. Thus, GLP-1 might be in line with natriuretic peptides or interleukin 10 (IL-10), which are upregulated following inflammatory stimuli and predict adverse clinical outcomes in patients with myocardial infarction and HF, respectively, but remain protective as endogenous counter-regulatory factors [143,144,145,146].

This hypothesis seems to be supported by clinical data on GLP-1 and its receptor agonists (GLP-1 RAs) in ACS patients. In a pilot study with a small number of patients with and without T2DM, 72 h of intravenous GLP-1 infusion to patients with ACS undergoing percutaneous revascularization improved left ventricular ejection fraction (LVEF) and regional wall motility [147]. Similar evidence with GLP-1RAs suggests an improved salvage of myocardium at risk for necrosis with intravenous exenatide [148] and a reduced infarct size with subcutaneous exenatide [149]. Liraglutide treatment reduced the resulting necrotic area [150] and improved LVEF after pPCI for STEMI [151] and non-STEMI [152]. These effects of liraglutide led to benefit in the group of patients with atherosclerotic CV disease (ASCVD), but not in that with multiple CV risk factors (MCVRF) alone [153].

In the DPP-4 inhibitors (DPP-4Is) family, sitagliptin has been tested in BEGAMI trial. The aim of this study was to validate the hypothesis that sitagliptin could improve beta-cell function in patients with recent ACS plus newly diagnosed IGT or T2DM, and that such treatment may be safely initiated soon after the coronary event [154]. In effects, sitagliptin improved beta-cell function and glucose perturbations in patients with ACS and newly diagnosed glucose abnormalities.

It has also been reported that sitagliptin improved LVEF and regional contractility during dobutamine-induced stress, with a preferential effect in ischemic segments of the heart [155]. However, unfortunately, the beneficial CV effects attributed to DPP-4 inhibition in mechanistic studies cannot easily be reconciled with the neutral results of large-scale clinical trials [156,157,158]. In fact, inhibition of DPP-4 neither increased nor decreased the risk of the combined major adverse CV event (MACE) outcome, which includes CV death, myocardial infarction or stroke [136].

Controversial data are available about the potential benefit of GLP-1 RAs and DDP-4Is in ACS patients with previous or developing HF. Harmony Outcomes was the only trial that demonstrated prevention of HF events in at-risk patients with T2DM. In this study, albiglutide was associated with a statistically significant reduction in HF hospitalisation [159,160]. Conversely, liraglutide, exenatide and semaglutide showed a consistent reduction in MACE and CV all-death, but there was no positive effect on outcome of patients with HF [161,162,163]. Given the results from RCTs and the pharmacological properties of GLP-1 RAs that increase the heart rate, the safety of their use in ACS patients with HF with reduced ejection fraction remains uncertain. Similarly, the DPP-4 I saxagliptin significantly increased the risk for hospitalization for congestive HF in the SAVOR TIMI 53 trial [157].

In conclusion, further data are needed to define whether incretin-based therapies may be used to improve the prognosis of non-diabetic patients with SIH at hospital admission for ACS. Thus far, GLP-1 RAs showed more promising result than DPP-4Is to address this aim in patients without HF.

### 5.5. Sodium-Glucose Co-Transporter 2 Inhibitors (SGLT-2Is)

Sodium-glucose co-transporter 2 inhibitors (SGLT-2Is), also known as glifozins, are a novel class of antidiabetic drugs which reduce the reabsorption of glucose and sodium from the proximal convoluted tubules, resulting in glycosuria and natriuresis properties [164]. The EMPA-REG OUTCOME trial reported the CV benefits of empagliflozin by significantly decreasing the incidence of hospitalization associated with HF, CV-cause and all-cause death rate in diabetic patients with ASCVD [165]. Although the results from this trial found no significant differences in the rate of ACS between group receiving treatment and placebo [165], there was a possible link between SGLT-2Is and cardioprotective effects attenuating ACS severity as emerged by animal and human studies [166,167,168]. Four-week pretreatment with dapagliflozin could decrease infarct size in rats with obese-IR which underwent myocardial ischemia–reperfusion injury (MIRI) [168]. In a chronic myocardial infarct model in rats, dapagliflozin treatment beginning one day after left anterior descending coronary artery ligation could decrease myofibroblast infiltration and myocardial fibrosis [167]. More recently, in a murine model of MIRI, dapagliflozin given pre-ischemia conferred the maximum level of cardioprotection quantified through the decrease in arrhythmia, attenuated infarct size, decreased cardiac apoptosis and improved left ventricular function, whereas dapagliflozin given during ischemia also showed cardioprotection, but at a lower level of efficacy [166]. In humans, EMPA-HEART trial has demonstrated that empagliflozin decreased left ventricular mass (assessed by cardiac magnetic resonance) after six months in patients with T2DM and either previous coronary revascularization or history of ACS, thus suggesting that SGLT2is may lead to improvement in LVR after ACS and secondarily decrease supply–demand mismatch ischemia [169].

Consistently with results of pre-clinical studies, an analysis from the DECLARE-TIMI 58 trial showed lower rates of MACE with dapagliflozin in patients with previous ACS, whereas there was no apparent reduction in either patients with established ASCVD but no previous ACS or patients with MCVRF alone [170]. Furthermore, in patients with previous ACS, dapagliflozin consistently reduced the composite endpoint of CV death or hospitalisations for HF compared to patients without previous ACS [170]. This benefit seemed to be higher the closer it was the acute event. These findings were in accord with a previous meta-analysis reporting that SGLT2-Is reduced the risk of MACE in patients with ASCVD, but not in those with MCVRF alone [171]. A recent subanalysis from EMPAREG OUTCOMES trial confirmed these data [172].

Therefore, SGLT-2Is showed more promising results than GLP-1 RAs and DPP-4Is in reducing MACE, CV death and hospitalizations for HF in patients with previous ACS. However, further RCTs are needed to validate this hypothesis in acute ACS patients. To this purpose, a phase 3b trial aimed to enroll patients with ACS and characteristics suggestive of severe myocardial necrosis has been very recently proposed [173]. The primary endpoint will be the impact of empagliflozin on changes in N-terminal pro-brain natriuretic peptide (NT-proBNP) within six months after ACS, whereas the secondary endpoints will include hospitalization rate due to HF and all-cause mortality.

## 6. Multitargeted Therapeutic Strategy for SIH in Non-Diabetic patients with ACS

The difficulty to choose a uniform therapeutic strategy for SIH in ACS non-diabetic patients derives from the still lacking individuation of optimal glycaemic targets to improve the outcomes and from the multitude of determinants of this condition.

Most followed consensus documents recommend maintaining glucose in the 140–180 mg/dL (7.78–10 mmol/L) range for patients with SIH in the intensive care unit (ICU) [174,175]. Consistently, NICE recommendations suggest to “manage hyperglycaemia in patients admitted to hospital for ACS by keeping blood glucose levels below 198 mg/dL (11.0 mmol/L) while avoiding hypoglycaemia” [176]. A more recent observational study identified the value of 140 mg/dL (7.78 mmol/L) as the threshold for clinically acceptable admission glucose levels in non-diabetic patients with ACS. In fact, although mildly elevated admission plasma glucose levels (60–140 mg/dL, corresponding to the range 3.33–7.78 mmol/L) are associated with increased one-year mortality, they are probably not the cause for the increased mortality risk, but rather a marker for a high risk population [177]. The ESC guidelines on management of non-STEMI/STEMI state that “it is reasonable to manage hyperglycaemia by maintaining a blood glucose concentration <200 mg/dL (11.0 mmol/L), but absolutely avoid hypoglycaemia” [178,179]. In accord with these findings, in an ACS patient naive to antidiabetic drugs and without known diabetes, we usually start the glucose-lowering therapy when glycaemic values are above 180 mg/dL (10 mmol/L) and try to maintain them within 140–180 mg/dL (7.78–10 mmol/L) range. In our experience, this range is useful to reduce the risk of hypoglycaemia and the incidence of glucose variability. When hypoglycaemia is severe, it can cause death from CV or neural events [180]. Consistently, glucose variability has emerged as a key variable in predicting outcome in critically ill patients. In fact, it has been demonstrated that glucose variability is a stronger predictor of mortality in ICU patients without a history of diabetes compared to those with a history of diabetes [181].

**Figure 2:**
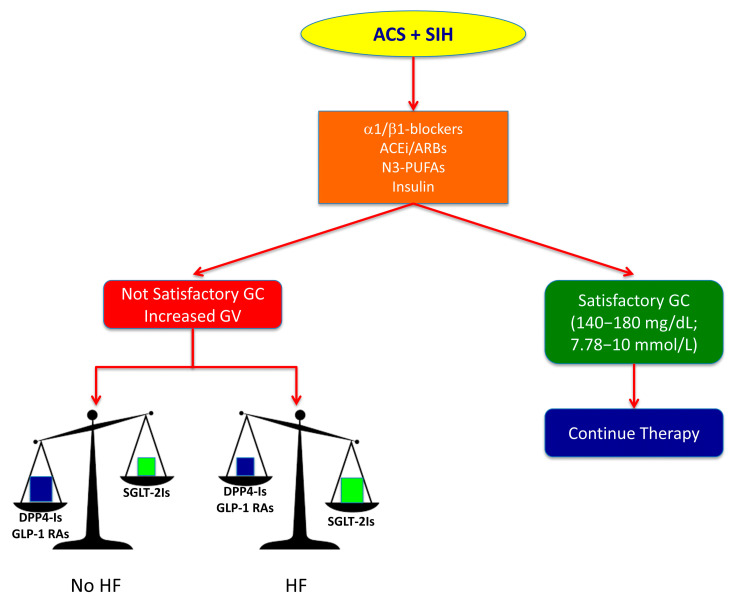
Proposal for a multitargeted therapeutic strategy to improve the prognosis in non-diabetic patients with stress-induced hyperglycaemia (SIH) at admission for acute coronary syndrome (ACS). Pharmacological therapy for SIH in non-diabetic ACS patient should start with administration of α1/β1-blockers, ACE inhibitors (ACEi) or angiotensin receptor blockers (ARBs) and n3-polyunsaturated fatty acids (n3-PUFAs) in addition to insulin. If this strategy reaches an efficient glycaemic control (GC) within the recommended range (140–180 mg/dL; 7.78–10 mmol/L), it may be continued without any change. Conversely, if glycaemic values are not satisfactory and glycaemic variability (GV) increases, we can implement this therapy with dipeptidyl peptidase-4 inhibitors (DPP-4Is) or glucagon like peptide-1 receptor agonists (GLP-1 RAs) in patients without signs of or risk factors for heart failure (HF), whereas sodium-glucose co-transporters 2 inhibitors (SGLT-2Is) must be preferred in patients with signs of or risk factors for HF.

Contemporarily, the multifactorial pathogenesis of SIH in non-diabetic patients with ACS suggests the use of a multitargeted therapeutic strategy aimed to counteract molecular mechanisms responsible for elevated glycaemic levels and CV injury (Figure 2). Highly β1-selective blockers (nebivolol), RAAS modulators (ACE-inhibitors and ARBs) and n-3 PUFAs have shown an important role in counteracting adrenergic overdrive, FFAs concentration increase and RAAS overactivity, thereby contributing to improve IR in hyperglycaemic non-diabetic patients. All these drugs are recommended as therapeutic options in European and American guidelines [182,183]. Insulin is also required, in the first instance, for treatment of SIH, as reported in NICE recommendations [176], but it is frequently not enough to control acute hyperglycaemia because it is not able to suppress glucagon secretion, which is one the principal causes of SIH. The lack of a satisfactory glycaemic control may move to increase insulin dosage thereby inducing hypoglycaemia and glycaemic variability. The prevalence of insulin-induced hypoglycaemia in SIH clinical trials can exceed 6%, even when more conservative glucose levels are targeted [180]. 

In this context, the role of GLP-1 RAs may become relevant. Liraglutide [184] and semaglutide [185] displayed the largest difference, relative to patients treated with placebos, in glycohemoglobin (HbA1c) which has been identified as an important potential mediator of CV benefits induced by GLP-1 RAs. In particular, the normalization of HbA1c levels is related to the reduction of MACE and non-fatal stroke [186]. Furthermore, liraglutide and semaglutide determine a substantial weight loss [184,185] and a better control of systolic blood pressure [184,185]. 

Interestingly, the magnitude of changes in HbA1c, body weight and systolic blood pressure elicited by liraglutide and semaglutide were smaller and maintained for shorter periods of time than would be predicted to be sufficient to elicit CV benefits based on previous analyses of glucose-lowering strategies [187,188]. Thus, it appears likely that traditional risk factor modification alone cannot explain the overall benefits observed, but rather additional mechanisms may be operative, most likely including direct effects in the CV system. In particular, GLP-1 has anti-inflammatory and antiatherogenic properties [148,189,190,191,192,193], and it counterbalances oxidative stress induced by both hyperglycaemia and hypoglycaemia in T1DM [194]. Furthermore, incretin treatment may prevent atherosclerosis progression or plaque vulnerability in T2DM patients [195]. In addition, because chronic kidney disease (CKD) is a frequent co-morbidity in ACS patients, it is noteworthy to remark that liraglutide, dulaglutide and semaglutide may be used in advanced CKD at any level of glomerular filtration rate without dose adjustment [196].

It is important to underline that, due to the delay in gastric emptying, GLP-1 RAs may induce gastrointestinal adverse effects, such as nausea, vomiting and diarrhoea. However, the risk of gastrointestinal side effects was slight and significantly lower with long-acting GLP-1 RAs [197].

We believe that a combined therapy of long-acting GLP-1 RAs (liraglutide, dulaglutide and semaglutide) with basal insulin may be the right choice in ACS patients with SIH and without HF signs. In fact, when a GLP-1 RA is added to basal insulin, the combination is as effective as an intensified insulin regime in terms of HbA1c control, but with a much lower risk of hypoglycaemia and weight gain [198]. In accord with our thought, a recent meta-analysis described the advantages of combining long-acting GLP-1 RAs (compared to short-acting GLP-1 RAs) with basal insulin [197]. Interestingly, not only HbA1c was more significantly lowered and HbA1c targets were achieved in a higher proportion of patients, but also fasting plasma glucose concentrations and body weight were controlled better with long-acting GLP-1 RAs [197].

The 2020 European Society of Cardiology guidelines recommend GLP-1RA as a first-line therapy in drug-naïve patients with T2DM and CV disease [183]. Consistently, the 2020 update to the American Diabetes Association Standards of Medical Care in Diabetes recommends that patients with established ASCVD or with indicators of high ASCVD risk preferentially receive a GLP-1RA with demonstrated CV benefit independent of glycaemic control [182].

Nevertheless, because controversial results about benefits of GLP-RAs and DPP-4Is combination in patients with HF, such drugs are not recommended in this setting. Additional analyses from EMPA-REG OUTCOME revealed that the CV benefit was gained by those with and without HF at baseline, the latter comprising ≈ 10% of the study cohort [199]. Consistently, a meta-analysis suggested relevant benefits on reducing the composite of HF hospitalization or CV death regardless of existing ASCVD or a history of HF [171]. The CV benefits of SGLT-2Is are mostly unrelated to the extent of glucose lowering. The rapid separation of placebo and active arms in the reduction in HF hospitalizations indicates that the beneficial effects achieved in these trials are more likely the result of a reduction in HF-associated events. They could involve effects on haemodynamic parameters, such as reduced plasma volume, direct effects on cardiac metabolism and function or other CV effects [200,201,202,203].

The effect of SGLT-2Is addition to insulin therapy on glycaemic control is still uncertain. All available data come from small sample-size studies. Canagliflozin improved blood glucose changes in T2DM using insulin [204]. A more recent clinical trial showed that dapaglifozin effectively decreases glucose levels after 12 weeks of treatment in participants with T2DM receiving insulin treatment, even if no effect was observed in glucose variability. However, significant reductions in HbA1c, glycated albumin and mean glucose levels derived by continuous glycaemic monitoring were observed in the dapagliflozin group compared with the placebo group [205]. In addition, although combination therapy of dapagliflozin and insulin was not superior in glucose fluctuation to DPP-4Is and insulin association, it in part provided favourable effects on metabolism in patients with T2DM treated with insulin therapy [206].

These results allow speculating that a combined therapy of SGLT-2Is with basal insulin has to be preferred in ACS patients with SIH and HF. The last European guidelines stated that SGLT-2Is are potentially of particular benefit in patients who exhibit HF or who are at high risk for HF [183]. However, this finding has to be yet validated in the setting of ACS patients with SIH.

At discharge, according to NICE recommendations [176], we suggest to discriminate between an undiagnosed diabetes or a real SIH on the basis of HbA1c values in order to decide whether continuing, modifying or stopping hypoglycaemizing therapy.

## 7. Conclusions

SIH at admission for an ACS is associated with a less favourable outcome, especially in patients without known diabetes. This finding is principally dependent on the increased inflammation, pro-thrombotic and pro-aggregant states and direct CV injury determined by acute hyperglycaemia. Unfortunately, insulin treatment in these subjects did not show positive results in reduction of mortality. This is likely because SIH in ACS, compared to known diabetes, represents an epiphenomenon of other pathological conditions, such as adrenergic and renin-angiotensin system overactivity, hyperglucagonaemia, increase of circulating FFAs and pancreatic beta-cell dysfunction, that are not completely reversed by insulin therapy and so worsen the prognosis. Thus, the target of pharmacological therapy in this setting of patients is represented by the achievement of a satisfactory glucose control through the improvement of IR and antagonism of hyperglycaemizing pathways.

The use of highly β1-selective blockers (nebivolol), RAAS modulators (ACE-inhibitors and ARBs) and n-3 PUFAs may counteract adrenergic overdrive, FFAs concentration increase and RAAS overactivity, thereby contributing to improve IR. Consistently, a combined therapy of long-acting GLP-1 RAs and basal insulin may significantly lower HbA1c levels and reduce the risk of hypoglycaemia in patients without HF, whereas SGLT-2Is may significantly decrease HF incidence and hospitalization for HF. Therefore, a multitargeted therapeutic strategy seems to be required to improve the prognosis in ACS non-diabetic patients with SIH. Further RCTs are still needed to validate this hypothesis.

## Figures and Tables

**Table 1:** Synopsis of randomized clinical studies designed to compare the effect of intensive glycaemic control by insulin versus standard therapy in populations comprising a large percentage of non-diabetic patients with acute hyperglycaemia at admission for acute coronary syndrome.

Clinical Trial (Year)	Number of Patients	Study Population	Admission Glycaemia	Specific Glycaemic Target	Reached Glycaemic Target (Intervention vs. Control)	Primary Endpoint	Result
DIGAMI-1 (1995)	620	ACS	≈280 mg/dL (15.56 mmol/L)	126–180 mg/dL (7–10 mmol/L) in acute phase 90–126 mg/dL (5–7 mmol/L) post-recovery	173 mg/dL (9.61 mmol/L) vs. 211 mg/dL (11.72 mmol/L) during first 24 h	Intra-hospital Mortality	Neutral at 3 months
DIGAMI-2 (2005)	1253	ACS	≈229 mg/dL (12.72 mmol/L)	126–180 mg/dL (7–10 mmol/L) in acute phase 90–126 mg/dL (5–7 mmol/L) post-recovery	164 mg/dL (9.11 mmol/L) vs. 180 mg/dL (10 mmol/L) during first 24 h	Intra-hospital Mortality	Neutral at 3 months
HI-5 (2006)	244	ACS	≈198 mg/dL (11 mmol/L)	≥140 mg/dL (7.78 mmol/L)	149 mg/dL (8.28 mmol/L) vs. 162 mg/dL (9 mmol/L) during first 24 h	Intra-hospital Mortality	Neutral at 3 months
Marfella (2009)	50	ACS (CABG)	≥140 mg/dL (7.78 mmol/L)	80–140 vs. 180–200 mg/dL (4.44–7.78 vs. 10–11.11 mmol/L)	163 mg/dL (9.06 mmol/L) vs. 192 mg/dL (10.67 mmol/L)	LVEF, Oxidative Stress, Apoptosis	↑LVEF↓Oxidative Stress and Apoptosis
Marfella (2012)	50	STEMI (CABG)	≥140 mg/dL (7.78 mmol/L)	80–140 vs. 180–200 mg/dL (4.44–7.78 vs. 10–11.11 mmol/L)	161 mg/dL (8.94 mmol/L) vs. 194 mg/dL (10.78 mmol/L) vs. 182 mg/dL (10.11 mmol/L)	Myocardial Regeneration	↑Myocardial Regeneration
Marfella (2013)	194	STEMI(pPCI)	≥140 mg/dL (7.78 mmol/L)	80–140 vs. 180–200 mg/dL (4.44–7.78 vs. 10–11.11 mmol/L)	144 mg/dL (8 mmol/L) vs. 201 mg/dL (11.17 mmol/L)	Myocardial Salvage	↑Myocardial Salvage
Marfella (2012)	165	STEMI(pPCI)	≥140 mg/dL (7.78 mmol/L)	80–140 vs. 180–200 mg/dL (4.44–7.78 vs. 10–11.11 mmol/L)	145 mg/dL (8.06 mmol/L) vs. 191 mg/dL (10.61 mmol/L)	ISR	↓ISR
RECREATE (2012)	287	STEMI (pPCI)	≥144 mg/dL (8 mmol/L)	90–117 mg/dL (5–6.5 mmol/L) vs. standard therapy	117 mg/dL (6.5 mmol/L) vs. 143 mg/dL (7.94 mmol/L)	Glycaemia, Intra-hospital Mortality	↓Glycaemia, Intra-hospital Mortality
BIOMArKS2 (2013)	280	ACS	≥140 mg/dL (7.78 mmol/L)	85–110 mg/dL (4.72–6.11 mmol/L; day), 85–139 mg/dL (4.72–7.72 mmol/L; night) vs. <288 mg/dL (16 mmol/L)	112 mg/dL (6.22 mmol/L) vs. ≈130 mg/dL (7.22 mmol/L)	Intra-hospital Mortality, Re-Infarction	↑Intra-hospital Mortality, Re-Infarction

ACS, acute coronary syndrome; STEMI, ST-elevation myocardial infarction; pPCI, primary percutaneous coronary intervention; LVEF, left ventricular ejection fraction; ISR, intra-stent restenosis; ↑ indicates an increase; ↓ indicates a decrease.

**Table 2:** Synopsis of randomized clinical studies designed to compare the effect of glucose-insulin-potassium (GIK) versus standard therapy, independently on achievement of a specific glycaemic target, in populations comprising a large percentage of non-diabetic patients with acute hyperglycaemia at admission for acute coronary syndrome (ACS).

Clinical Trial (Year)	Number of Patients	Study Population	Admission Glycaemia	Specific Glycaemic Target	Reached Glycaemic Target (Intervention vs. Control)	Primary Endpoint	Result
ECLA-GIK (1998)	407	ACS	140 ± 15 mg/dL (7.78 ± 0.83 mmol/L; both GIK groups) vs. 143 ± 15 mg/dL (7.94 ± 0.83 mmol/L)	-	122 ± 7 mg/dL (6.78 ± 0.39 mmol/L; both GIK groups) vs. 135 ± 5 mg/dL (7.5 ± 0.28 mmol/L)	In-hospital mortality	Similar In-hospital mortality
GIPS (2003)	940	STEMI	153 mg/dL (8.5 mmol/L) in both groups	-	139 ± 10 mg/dL (7.72 ± 0.56 mmol/L) vs. 146 ± 10 mg/dL (8.11 ± 0.56 mmol/L)	30 day-Mortality	Similar 30 day-Mortality
GIPS-2 (2006)	889	STEMI (Killip Class I)	153 ± 50.4 mg/dL (8.5 ± 2.8 mmol/L) vs. 149.4 ± 45 mg/dL (8.28 ± 2.5 mmol/L)	-	-	30 day-Mortality	Similar 30 day-Mortality
CREATE-ECLA (2005)	20,201	STEMI	162 mg/dL (9 mmol/L) in both groups	-	187 mg/dL (10.39 mmol/L) vs. 148 mg/dL (8.22 mmol/L)	30 day-Mortality	Similar 30 day-Mortality
OASIS-6 GIK (2007)	2748	STEMI(14.9% vs. 14%)	-	-	-	30 day-Mortality	Similar 30 day-Mortality
IMMEDIATE (2012)	911	ACS	-	-	-	Progression to AMI, 30 day-Mortality	Similar Progression to AMI and 30 day-Mortality

## Data Availability

No new data were created or analyzed in this study. Data sharing is not applicable to this article.
